# Protein–lipid architecture of a cholinergic postsynaptic membrane

**DOI:** 10.1107/S2052252520009446

**Published:** 2020-07-28

**Authors:** Nigel Unwin

**Affiliations:** a MRC Laboratory of Molecular Biology, Francis Crick Avenue, Cambridge Biomedical Campus, Cambridge CB2 0QH, United Kingdom

**Keywords:** nicotinic acetyl­choline receptor, postsynaptic membrane, cholesterol microdomain, membrane protein, helical image reconstruction

## Abstract

The cholinergic postsynaptic membrane is an acetyl­choline receptor-rich membrane mediating fast chemical communication at the nerve–muscle synapse. Here, cryo-EM is used to examine the protein–lipid architecture of this membrane in tubular vesicles obtained from the (muscle-derived) electric organ of the *Torpedo* ray. As reported earlier, the helical arrangement of the protein component of the vesicles facilitates image averaging and enables us to determine how cholesterol and phospho­lipid molecules are distributed in the surrounding matrix, using headgroup size as a means to discriminate between the two kinds of lipid. It is shown that cholesterol segregates preferentially around the receptors in both leaflets of the lipid bilayer, interacting robustly with specific transmembrane sites and creating a network of bridging microdomains. Cholesterol interactions with the receptor are apparently essential for stabilizing and maintaining its physiological architecture, since the transmembrane structure contracts, involving displacements of the helices at the outer membrane surface by ∼2 Å (1–3 Å), when this lipid is extracted. The microdomains may promote cooperativity between neighbouring receptors, leading to an enhanced postsynaptic response.

## Abstract 2

Cryo-EM reconstruction of the native *Torpedo* membrane reveals that cholesterol segregates preferentially around nicotinic acetyl­choline receptors, interacting robustly with specific transmembrane sites and creating a network of bridging microdomains. The microdomains may promote cooperativity between neighbouring receptors, leading to an enhanced postsynaptic response.

## 1. Introduction   

Rapid communication in the nervous system takes place at the chemical synapse, which acts as a fundamental unit transmitting electrical impulses between nerves and their target cells, forming circuits and underpinning virtually all functions of the brain. The postsynaptic membrane, apposing the pre-synaptic nerve terminal, is where transmitter-gated ion channels are concentrated. These fast-acting proteins respond transiently to pre-synaptic release of neurotransmitter, opening cation- or anion-selective pathways across the membrane to effect a change in membrane potential. The postsynaptic membrane, by eliciting this response, plays a critical role in determining the efficacy and speed of synaptic transmission. Furthermore, its protein–lipid composition, organization and size are subject to modification by physiological events, making it an important mediator of neuronal plasticity.

**Figure 1:**
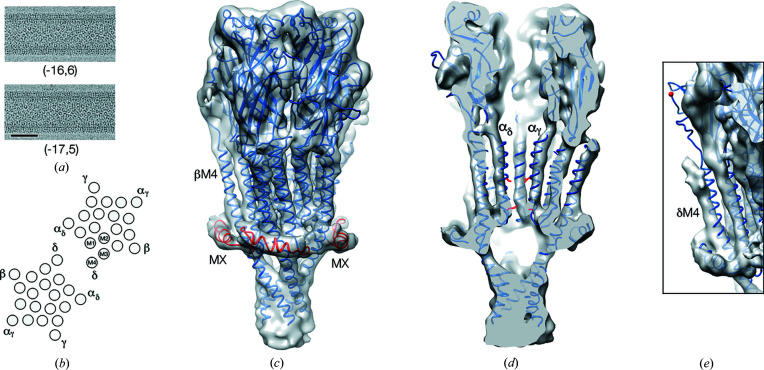
Structure of the ACh receptor in *Torpedo* postsynaptic membrane. (*a*) Micrographs of the tubular vesicles analysed in this study. The scale bar represents 500 Å. (*b*) Arrangement of subunits and TM helices in receptor dimers, forming the helical asymmetric unit. (*c*) The 5.8 Å density map and superimposed 2.7 Å structure of the *Torpedo* receptor (PDB entry 6uwz) obtained from detergent-solubilized protein complexed with α-bungarotoxin. MX helices are shown in red. (*d*) A sectional view through the map and superimposed model, showing details of the central transmembrane pore (functionally important amino acid residues on the α-subunit pore-lining M2 helices, 9′Leu, uppermost, and −1′Glu, are shown in red). (*e*) Part of the δ subunit involved in dimer formation, showing mismatch with the model (δ–δ disulfide bridge-forming cysteine shown in red). The C-terminal portion of δ, beyond M4, is not seen in the density map and is probably flexible (accounting for the variable configurations of isolated dimers; Rahman *et al.*, 2020 ▸).

The cholinergic membrane of the nerve–muscle synapse is the best-understood postsynaptic membrane and its principles of operation serve to illuminate principles pertaining to the more complex synapses of the central nervous system. Here, we analyse by cryo-EM the architecture of this membrane in vesicles isolated from the (muscle-derived) electric organ of the *Torpedo* ray, with view to defining the protein–lipid interplay required to achieve an optimal neurotransmitter response. The vesicles are in the form of ∼760 Å diameter tubes, with the constituent acetyl­choline (ACh)-receptor ion channels embedded in their natural cholesterol-rich phospho­lipid bilayer, and packed side-by-side in a configuration recapitulating their organization at the synapse [Fig. 1 ▸(*a*); Heuser & Salpeter, 1979 ▸; Cartaud *et al.*, 1981 ▸; Brisson & Unwin, 1984 ▸]. As reported earlier (Unwin, 2017 ▸), the helical arrangement of the protein component of the vesicles facilitates image averaging and enables us to determine how cholesterol and phospho­lipid molecules are distributed in the surrounding matrix, using headgroup size as a means to discriminate between the two kinds of lipid.

We find that cholesterol segregates preferentially around the receptors in both leaflets of the lipid bilayer, interacting robustly with specific transmembrane sites and creating a network of bridging microdomains. The structural evidence suggests that cholesterol interactions with the receptors are essential for maintaining their physiological architecture and that the microdomains may contribute to enhancement of the postsynaptic response.

## 2. Methods   

### 2.1. Specimen preparation   

Tubular cholinergic membranes were prepared from the electric organ of a freshly killed *Torpedo* ray (*Torpedo marmorata*). The tissue was homogenized to release ACh receptor-rich vesicles, which were purified by centrifugation and converted into tubes by incubation in 100 m*M* sodium cacodylate, 1 m*M* calcium chloride, pH 7.0 at 17°C (Kubalek *et al.*, 1987 ▸). Aliquots (3.8 µl) of the tube-containing solution were applied to holey carbon support grids and blotted to retain the specimens in a thin aqueous film before plunging into liquid nitro­gen-cooled ethane.

### 2.2. Cryo-EM data collection and image processing   

The specimens were imaged with an FEI Titan Krios transmission electron microscope incorporating a 70 µm diameter objective aperture and operating in nanoprobe mode at an accelerating voltage of 300 kV. Micrographs were recorded in integrating mode on a Falcon 3 4096 pixel direct-electron detector after searching for straight ice-embedded tubes spanning holes in the carbon support film. Defocus values ranged from −1 to −2.8 µm. The calibrated pixel size on the specimen was 1.34 Å. The total dose was 40 e Å^−2^ fractionated from 79 frames. Micrograph frame stacks were drift corrected and dose weighted using *MotionCor2* (Li *et al.*, 2013 ▸). Local contrast-transfer functions were estimated from the aligned non-dose-weighted micrographs using *Gctf* (Zhang, 2016 ▸). All subsequent image-processing steps were performed on the dose-weighted micrographs using the single-particle method of helical reconstruction (Egelman, 2000 ▸, Sachse *et al.*, 2007 ▸), as implemented in *RELION* (Scheres, 2012 ▸; He & Scheres, 2017 ▸).

We analysed tubes belonging to the (−16, 6) and (−17, 5) helical families [Fig. 1 ▸(*a*); Toyoshima & Unwin, 1990 ▸]. These tubes have dihedral D2 and D1 symmetry, respectively, and were treated as two-start and single-start helices incorporating the physiological δ–δ subunit dimer of receptors [Fig. 1 ▸(*b*); Chang & Bock, 1977 ▸; Miyazawa *et al.*, 2003 ▸] as the helical asymmetric unit. The two kinds of tube have equivalent surface lattices but the surface lattice of the (−17, 5) tubes is rotated by 3.6° relative to that of the (−16, 6) tubes.

The image-processing workflow is summarized in Fig. S1 of the Supporting information. Micrographs of tubes, with appropriate helical symmetry, were selected by inspection of Fourier transforms of the images. Tubes from the selected micrographs were divided into overlapping segments using a box size of 1024 pixels and an inter-box spacing of 60 pixels, and were binned initially times two. Reference-free two-dimensional classification applied to the extracted segments yielded ∼95% of sufficient quality for further processing. Three-dimensional classification was conducted in two rounds to obtain class averages characterized by distinct values for the helical parameters (twist and rise) and for tube radius. A large fraction of the box length (65%) was used to search for and impose helical symmetry in the first round, whereas a short fraction (10%) was used in the second. The first round sorted those segments with greatest sensitivity to variations in the helical parameters, whereas the second round, at higher resolution, was more discriminating for tube radius (see also Fig. S2). Heterogeneities associated with membrane tubes, such as variations in lipid content, crystalline disorder and thin ice-induced flattening, appeared to account for most of the segments eliminated by the two-step classification. The best class averages obtained in this way (12 for each family) were subject to a higher-resolution refinement, using re-extracted un-binned data and average values for the helical twist and rise, to yield the final reconstructions.

For each helical family, densities corresponding to single receptors were cut out at radially equivalent coordinates from the individual reconstructions, using a soft spherically capped cylindrical mask, and averaged [Fig. S3(*a*)]. Sectional views were cut out and averaged in the same way. Estimated resolutions [6.2 Å for both families; Fig. S3(*b*)] were based on comparison of single-receptor densities averaged over half-sets (*i.e.* 6 of the 12 individual reconstructions).

The 5.8 Å structure [Fig. 1 ▸; Fig. S3(*b*)] was obtained by averaging the reconstructions from each helical family after realignment to account for the 3.6° difference in lattice rotation. Negative *B* factors (*B* = −500 Å^2^) were applied to sharpen the ‘single-particle’ maps (Fernández *et al.*, 2008 ▸; Figs. 1 ▸, S4 and S5).

The model (PDB entry 6uwz; Rahman *et al.*, 2020 ▸) was fitted to the densities using *UCSF Chimera* (Pettersen *et al.*, 2004 ▸). Structural figures were prepared using both *UCSF Chimera* and *PyMOL* (DeLano, 2002 ▸).

## 3. Results   

We examined tubes belonging to two helical families [Figs. 1 ▸(*a*) and 1 ▸(*b*)]. In each family, the image processing (see *Methods* [Sec sec2]) yielded 12 class averages where features both of the receptor and the lipid bilayer were well resolved. The corresponding within-family reconstructions shared similar helical parameters (twist and rise) but varied in radius by as much as 8 Å [Fig. S2(*a*)]. Therefore, to determine the averaged densities of a single receptor and of stretches of membrane along the tube axis, we re-calculated the class averages imposing fixed (average) values for the helical parameters and then brought the individual reconstructions into local equivalence by radial realignment. Finally, a 5.8 Å density map of a single receptor was obtained by averaging the aligned densities from both kinds of tube.

### 3.1. Structure of receptor in its native membrane setting   

Fig. 1 ▸(*c*) shows the density map of a single receptor determined in this way, with a recently solved 2.7 Å structure of the *Torpedo* receptor (Rahman *et al.*, 2020 ▸) superimposed. Although the 2.7 Å model is derived from detergent-isolated monomeric protein, and is complexed with α-bungarotoxin, it contains the sub-membrane rim-forming helix MX, missing from earlier models, and provides a good fit to the densities in most regions of the map [Fig. 1 ▸(*c*)]. Exceptions include the upper portion of the pore, which in the model is more constricted [Fig. 1 ▸(*d*)] owing to a more compact arrangement of TM helices (Figs. S4 and S5), and the δ subunit [Fig. 1 ▸(*e*)], which in the membrane is linked to the δ subunit of a neighbouring receptor through a disulfide bridge (Chang & Bock, 1977 ▸).

**Figure 2:**
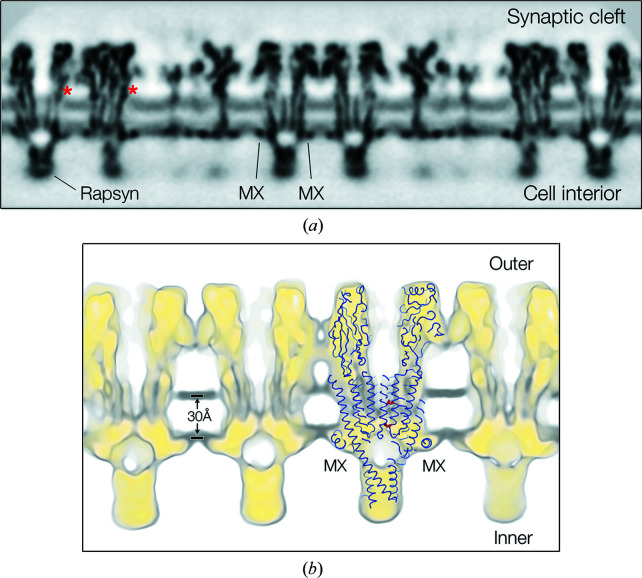
Cross sections showing ACh receptors in the context of the lipid bilayer. (*a*) A profile view from (−16, 6) tubes, displaying the whole membrane as it would appear *in situ*. The unassigned density at the base of the receptor most probably arises from the attached (but not helically ordered) protein rapsyn (Toyoshima & Unwin, 1988 ▸; Zuber & Unwin, 2013 ▸). Asterisks identify patches of weakened lipid density next to the protein surfaces in the outer leaflet attributed to cholesterol. (*b*) A 15 Å slab through the membrane in a similar orientation and superimposed model (PDB entry 6uwz), relating the structure to the phospho­lipid headgroup locations (dark grey). Amino acid residues 9′Leu and −1′Glu of the β subunit are shown in red. The continuous yellow-to-grey background spans densities ranging from 3.5–0.8σ.

Profile views [Fig. 2 ▸(*a*)] show details of the protein in the context of the lipid bilayer, highlighting in particular the involvement of MX. The strongly electron-scattering phospho­lipid headgroups give rise to bands of higher density (‘tram tracks’) on either side of the near-central low-density fluid portion of the hydro­phobic core. The strongest densities in the two leaflets, at the level of the phosphate moieties (Caspar & Kirshner, 1971 ▸; Franks, 1976 ▸), are only 30 Å apart [Fig. 2 ▸(*b*)], *i.e.* closer together than in most biological membranes (Gerle, 2019 ▸).

As Fig. 2 ▸(*b*) also indicates, the 9′Leu hydro­phobic gate of the receptor (White & Cohen, 1992 ▸; Labarca *et al.*, 1995 ▸), and the ring of negatively charged residues at the −1′ position, where cation selectivity and the size of the permeating ions are largely determined (Imoto *et al.*, 1988 ▸; Keramidas *et al.*, 2004 ▸; Cymes & Grosman, 2016 ▸), both lie within boundaries framed by the phospho­lipid headgroups.

### 3.2. Organization of cholesterol around the receptor   

The earlier study (Unwin, 2017 ▸) described patches in the outer leaflet of the bilayer, next to the protein surfaces, exhibiting weaker densities than the surrounding phospho­lipid-rich matrix. These patches were interpreted to consist of cholesterol because they contributed no measurable density at the level of the phospho­lipid headgroups, consistent with the fact that cholesterol exposes only a hydroxyl and would present no mass this far from the hydro­phobic core. Accordingly, the presence of cholesterol in the underlying leaflet was identified with discontinuities in the otherwise rather uniform densities arising from the phospho­lipid headgroups when viewed in profile [*e.g.* at asterisks, Fig. 2 ▸(*a*)] and with gaps among the phospho­lipid-headgroup densities in in-plane views.

**Figure 3:**
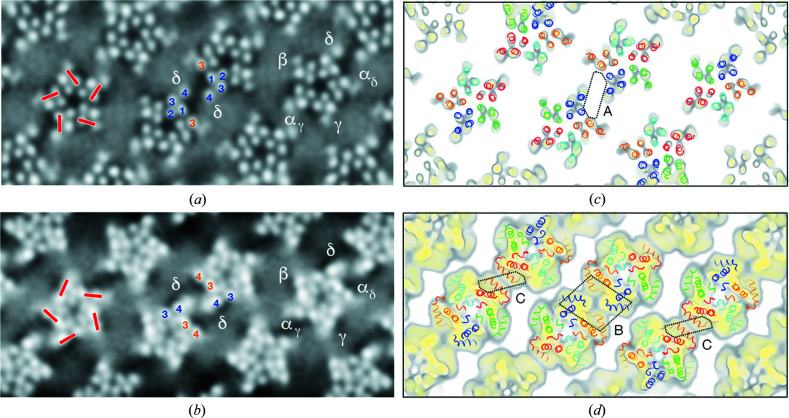
Organization of phospho­lipids (smooth grey areas) and cholesterol (darker patches) next to the transmembrane surfaces viewed in sections tangential to the tube axis. (*a*) At the level of the outer phospho­lipid headgroups, showing small cholesterol-rich patches next to M4, M1 and the adjacent M3 helices (red bars); also showing a larger patch (microdomain) bridging the central δ–δ dimer. (*b*) At the level of the inner phospho­lipid headgroups, showing cholesterol-rich patches next to the M3 and M4 helices (red bars); also showing two additional microdomains. (*c*), (*d*) Slabs encompassing the outer (*c*) and inner (*d*) headgroup regions, with structures superimposed to identify regions of the protein responsible for the features in (*a*) and (*b*). The microdomains A, B and C bridge dimer interfaces involving the δ, α_δ_/δ and α_γ_ subunits, respectively. Both (*a*) and (*b*) are shown in inverted contrast; the numbering 1–4 identifies TM helices M1–M4. Subunit colours: α_γ_, red; α_δ_, orange; β, green; γ, cyan; and δ, blue.

**Figure 4:**
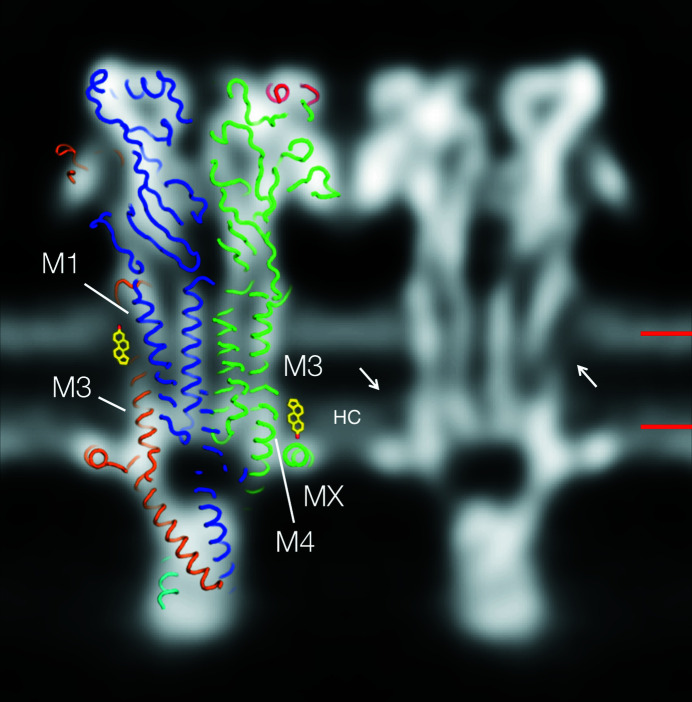
A section through the bilayer showing strong densities in the inner-leaflet hydro­phobic core attributable to tight packing of the phospho­lipid hydro­carbon chains. The densities (HC) extend into the hydro­phobic core about the same distance as would the sterol group of cholesterol. A matching slice through the model (PDB entry 6uwz) and two (manually inserted) sterol groups are superimposed on and next to one of the receptors to indicate the locations of two of the cholesterol sites identified in Fig. 3 ▸; arrows point to the equivalent sites on the other (twofold-related) receptor. Bars on the right indicate the levels of the sections in Figs. 3 ▸(*a*) and 3 ▸(*b*). The figure is shown in inverted contrast. The subunit colours are the same as those used in Fig. 3 ▸.

Fig. 3 ▸(*a*) shows a section in the plane of the outer phospho­lipid headgroups [subunit identifications in Fig. 3 ▸(*c*); level indicated in Fig. 4 ▸], confirming the small-patch/microdomain cholesterol organization that was described. The patches, including the central δ–δ microdomain [A, Fig. 3 ▸(*c*)], originate at equivalent locations on the subunits, each involving M4, M1 and the adjoining helix M3. In other words, cholesterol interacts in a robust and specific way with these three TM helices and ‘decorates’ them by variable amounts according to the size of patch. Other potential cholesterol-interacting sites on the lipid-facing surfaces are not seen, suggesting they would entail more transient associations that are blurred out by the image averaging.

A similar section through the inner leaflet, overlying MX [Figs. 3 ▸(*b*) and 3 ▸(*d*); level indicated in Fig. 4 ▸], shows a different distribution of patches, including two additional microdomains [bridging the α_δ_/δ pair at B and the α_γ_ pair at C; Fig. 3 ▸(*d*)]. The patches again originate at equivalent locations on each subunit but this time involving M4, M3 and the underlying helix MX. Hence, cholesterol interacts robustly with an alternative group of three helices in the inner leaflet.

Comparison of equivalent patches in either helical family indicates slight shape and size variability, suggesting slightly different lipid compositions comprising the two kinds of tube (Fig. S6). However, as would be expected, all patches have the same fixed locations against the protein surfaces, as just described.

Thus, considering the receptor as a whole, there are two regions on the subunits interacting robustly with cholesterol: one region implicating three adjacent TM helices in the outer leaflet, on the clockwise face; and the other region implicating two adjacent TM helices and the sub-membrane helix MX in the inner leaflet on the anticlockwise face. These regions are most commonly associated with small patches of cholesterol but evidently when brought into close proximity, like at dimer interfaces, they stabilize more extended patches, *i.e.* microdomains.

We note that the microdomains form only against the α and δ subunits. These subunits are major determinants of gating kinetics according to single-channel electrophysiological experiments (Sakmann *et al.*, 1985 ▸). The presence of the microdomains, and their locations, therefore support the notion that a distinct lipid environment might be responsible for the more rapid gating of ACh receptors that occurs in a synaptic setting (Neher & Sakmann, 1976 ▸).

### 3.3. Influence of cholesterol on neighbouring lipids   

The sections encompassing the phospho­lipid headgroup regions (Fig. 3 ▸) demonstrate a clear relationship between the chemical–physical make-up of the protein surfaces and the cholesterol component of the postsynaptic membrane. Does cholesterol, in turn, influence the organization of the phospho­lipids? Close inspection of features across the lipid bilayer, as in Fig. 4 ▸, shows that the strong densities normally attributable to just the phospho­lipid headgroup region can also extend further into the hydro­phobic core. In the inner leaflet, they penetrate the hydro­phobic core (labelled HC, Fig. 4 ▸) to roughly the same level as would a cholesterol sterol group. This is probably because of tight and ordered packing of the initial saturated portions of the hydro­carbon chains, imposed by the rigid ring structure, an effect of cholesterol observed originally in X-ray studies of myelin membranes (Caspar & Kirshner, 1971 ▸). As a result, the cholesterol sites identified in Fig. 3 ▸(*b*) are distinguishable by their slightly weaker densities not only at the level of the headgroups but also deeper into the hydro­phobic core.

## 4. Discussion   

This study extends an preliminary report on the protein–lipid architecture of the *Torpedo* postsynaptic membrane (Unwin, 2017 ▸). We recapitulate the physiological context by imaging and reconstructing the structure of the whole membrane, with the protein and lipid components organized as they are *in situ*. Although molecular details of the lipids are not yet visible using this approach, the reconstructed densities are sufficient to reveal an intricate network of protein–protein and protein–lipid interactions, and a segregated distribution of cholesterol next to particular protein surfaces. We find that cholesterol engages with specific transmembrane sites in both leaflets of the bilayer and also that it aggregates in patches around these sites, stabilized apparently by properties of the protein surfaces and its high concentration (40–46 mol% of the membrane lipids; Popot *et al.*, 1978 ▸; Gonzalez-Ros *et al.*, 1982 ▸; Rotstein *et al.*, 1987 ▸), which is not far from saturating amounts (50 mol% in lecithin bilayers; Lecuyer & Dervichian, 1969 ▸).

While not seen before in such detail, the partitioning of the lipids should not be surprising given the pivotal role of cholesterol in enabling the classical transitions of this ion channel between closed, open and desensitized states (Criado *et al.*, 1982 ▸; Ochoa *et al.*, 1983 ▸; Sunshine & McNamee, 1992 ▸; Ryan *et al.*, 1996 ▸; Rankin *et al.*, 1997 ▸; Hamouda *et al.*, 2006 ▸; daCosta & Baenziger, 2009 ▸). Most intriguing is the propensity of cholesterol to form networks bridging the ion-channel arrays, raising the possibility that it helps to coordinate their activity at the synapse.

### 4.1. Receptor–cholesterol complexes   

The finding that cholesterol occupies equivalent sites on all subunits around the receptor, in both leaflets of the bilayer [Figs. 3 ▸(*a*) and 3 ▸(*b*)], suggests that its intimate integration with the transmembrane architecture may be required for the protein to achieve full ion-channel function. Indeed, cholesterol is almost certainly needed to maintain the splayed-apart arrangement of the adjacent M4–M1–M3 helices in the outer leaflet. These helices are wide apart at the level of the phospho­lipid headgroups in the density map of the membrane-bound receptor (mean M4–M1 and M1–M3 separations of 14.2 and 12.8 Å, respectively) but they are ∼2 Å (1–3 Å) closer together in the structure of the solubilized protein, where cholesterol has been removed (Fig. S4). Moreover, the separation of the remaining lipid-facing pair of helices (M3–M4), where no cholesterol was detected [Fig. 3 ▸(*a*)], does not change (Fig. S5). Therefore, cholesterol must act to stabilize the splayed-apart architecture, presumably by wedging between the helices at their interfaces (Jones & McNamee, 1988 ▸; Brannigan *et al.*, 2008 ▸; Baier *et al.*, 2011 ▸).

In the inner leaflet, where the M3 and M4 helices are close together, cholesterol more likely attaches to their lipid-exposed faces, as occurs with the nicotinic α4β2 receptor (Walsh *et al.*, 2018 ▸) and is consistent with the profile view (Fig. 4 ▸). Assuming one cholesterol for each helix, and including the two putative outer-leaflet sites, a total of four cholesterols per receptor subunit would be required to achieve full functionality. This is one more cholesterol than has been estimated from biochemical data (Hamouda *et al.*, 2006 ▸).

Why are the cholesterol molecules so important in determining how the receptor operates? In the outer leaflet, a cholesterol-stabilized splayed-apart architecture may be crucial in enabling the rapid gating movements of the pore-lining M2 helices, which involve greatest displacements in the upper part of the pore (Unwin & Fujiyoshi, 2012 ▸). In the inner leaflet, the stiffness imposed by an encircling ring of rigid sterol groups would limit flexibility at the level of the −1′ position [Fig. 2 ▸(*b*)] and so may help make ion discrimination more precise.

### 4.2. MX: a molecular filter   

**Figure 5:**
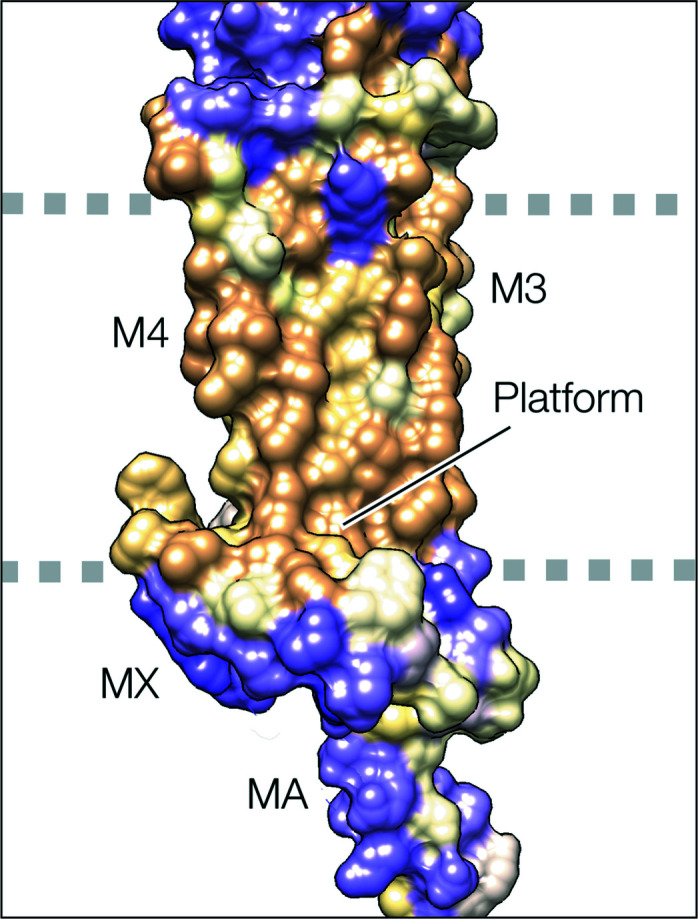
MX and adjoining helices in hydro­phobicity surface representation (from PDB entry 6uwz; α_δ_ subunit; tan, hydro­phobic; purple, polar). MX and the link to M3 are held by hydro­phobic contacts to the body of the receptor. Together they create an extensive hydro­phobic ‘platform’ facing the hydro­phobic core of the bilayer and a polar side facing the zwitterionic phospho­lipid headgroups [the dashed lines correspond to their positions indicated in Fig. 2 ▸(*b*)].

Studies with photoactivatable cholesterol analogues have demonstrated specific interactions of cholesterol with amino acids on M1, M3 and predominantly, M4 (Corbin *et al.*, 1998 ▸; Hamouda *et al.*, 2006 ▸), in agreement with the site assignments based on the reconstructed densities. However, it is clear from Fig. 3 ▸(*b*), showing microdomains occupying the overlying spaces, that the sub-membrane helix MX also promotes enrichment of cholesterol next to the receptor. Presumably, this is because MX penetrates the phospho­lipid headgroup region sufficiently to sterically exclude the large headgroups from the overlying membrane [Fig. 2 ▸(*b*)], while leaving room in the hydro­phobic core for the smaller cholesterol molecules to reside. As Fig. 5 ▸ shows, MX creates a favourable environment – a side-facing polar surface and a core-facing hydro­phobic ‘platform’ – to fulfil this role. In other words, MX has a design that would selectively accommodate cholesterols, supplementing those interacting more robustly with specific transmembrane sites and so further limiting flexibility of structure around the lower part of the pore.

### 4.3. Origin of microdomains   

**Figure 6:**
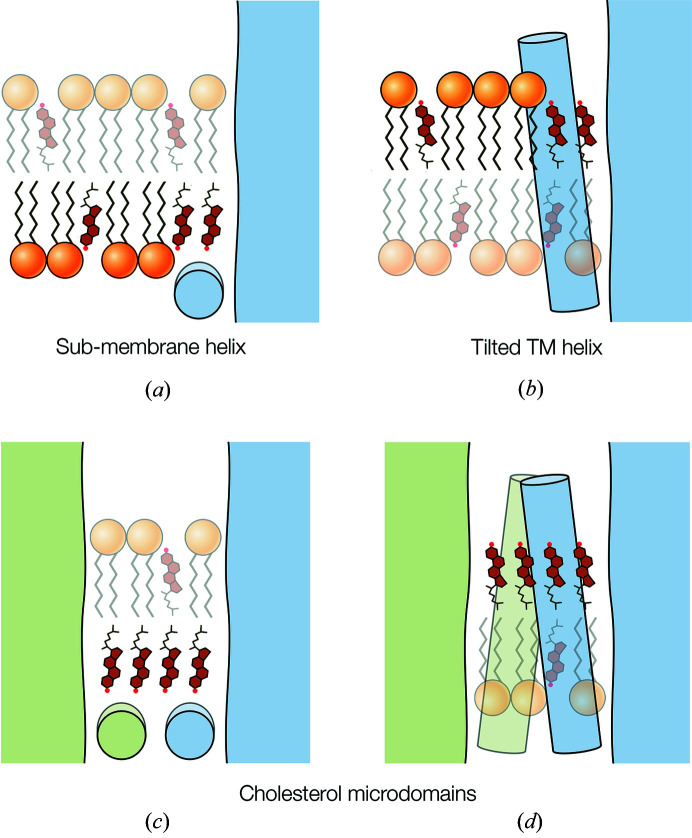
Protein motifs favouring extended patches of cholesterol. (*a*) A sub-membrane helix which sterically entraps cholesterol by disallowing access of large phospho­lipid headgroups. (*b*) A TM helix which has an affinity for cholesterol and tilts outward into the lipids. (*c*), (*d*) Interacting proteins (green and blue) which share these motifs, leading to the formation of bridging microdomains.

The aggregation of cholesterol into patches, in Fig. 3 ▸, can be considered a partial separation of cholesterol from the lipid matrix, catalyzed by the protein surfaces, which at higher (pathological) concentrations would materialize in the formation of crystals of cholesterol monohydrate (Varsano *et al.*, 2018 ▸). Two motifs of the receptor appear to promote such aggregation. The first is an amphipathic helix that penetrates the phospho­lipid group region but not the hydro­phobic core, exemplified by MX [Fig. 6 ▸(*a*)]. The second is a TM helix that interacts strongly with cholesterol and tilts away from the body of the protein [Fig. 6 ▸(*b*)], of which M4 of δ is the obvious example. When these motifs are brought into close apposition, as at dimer interfaces, the extent of the initial patch approximately doubles, hence creating a microdomain that bridges the two motifs. Microdomains B and C, where the MX helices come together [Figs. 3 ▸(*b*) and 3 ▸(*d*)], are of one type [Fig. 6 ▸(*c*)], and microdomain A, where the M4 helices of neighbouring δ subunits approach each other [Figs. 3 ▸(*a*) and 3 ▸(*c*)], is of the other type [Fig. 6 ▸(*d*)].

### 4.4. The δ–δ dimer and postsynaptic response   

The δ–δ dimer uniquely is bridged by cholesterol microdomains in both leaflets of the bilayer: one microdomain is associated with the tilted disulfide-linked M4 helices of the δ subunits and the other microdomain is associated with abutting MX helices of both δ and α_δ_ subunits. The connecting closely packed assemblies of sterol groups would confer rigidity on the points of contact between the two monomers. This is relevant because the monomer channels comprising *Torpedo* dimers, when reconstituted in cholesterol-rich planar membranes, open and close in synchrony, even in the absence of the disulfide bridge (Schindler *et al.*, 1984 ▸). Therefore, the imposed rigidity seems sufficient for the paired proteins to behave cooperatively.

Furthermore, with recordings made on isolated clusters, multiple synchronized gating (implicating at least three δ–δ dimers) is observed. It is plausible then that not only the δ–δ dimer microdomains (type A and B), but also the α_γ_–α_γ_ microdomain (type C) linking these dimers to each other, promote cooperative activity (see Fig. S6). The consequent network of cooperative interactions would be expected to increase the sensitivity to acetyl­choline and the magnitude of the postsynaptic response (Bray & Duke, 2004 ▸; Choi, 2014 ▸).

The extent of potential interacting networks in the highly specialized fish membrane is unlikely to be matched in the cholinergic postsynaptic membranes of muscle cells, where the channels also pack tightly but with less order. Nevertheless, the muscle receptors incorporate the same structural motifs (such as MX) and retain a similar ‘paired-ribbon’ organization as the *Torpedo* receptors (*e.g.* at the frog neuromuscular junction; Hirokawa & Heuser, 1982 ▸), so are likely to coordinate their ion-channel activity in a similar, if more limited, way.

## 5. Conclusions   

Cholesterol plays a vital role in supporting the function of the cholinergic postsynaptic membrane by stabilizing and maintaining the transmembrane architecture of its constituent ion channel, the nicotinic ACh receptor.

The surfaces of the ACh receptor harbour motifs designed to enrich local cholesterol concentrations, leading to the formation of microdomains which connect one receptor to the next.

The microdomains may promote cooperativity between neighbouring receptors, leading to an enhanced postsynaptic response.

## 6. Related literature   

The following reference is cited in the Supporting information for this article: Miyazawa *et al.* (1999) ▸. 

## Supplementary Material

Supporting figures. DOI: 10.1107/S2052252520009446/rq5005sup1.pdf

EMDB reference: ACh receptor, EMD-11239
