# Supplementary material for: Protein–lipid architecture of a cholinergic postsynaptic membrane
Source: IUCrJ. 2020 Jul 28;7(Pt 5):852–9. doi: 10.1107/S2052252520009446 (PMC7467168; doi:10.1107/S2052252520009446)
Supplement: Supplementary file 1 [file m-07-00852-sup1.pdf]

IUCrJ

Volume 7 (2020)

Supporting information for article:

Protein-lipid architecture of a cholinergic postsynaptic membrane

Nigel Unwin

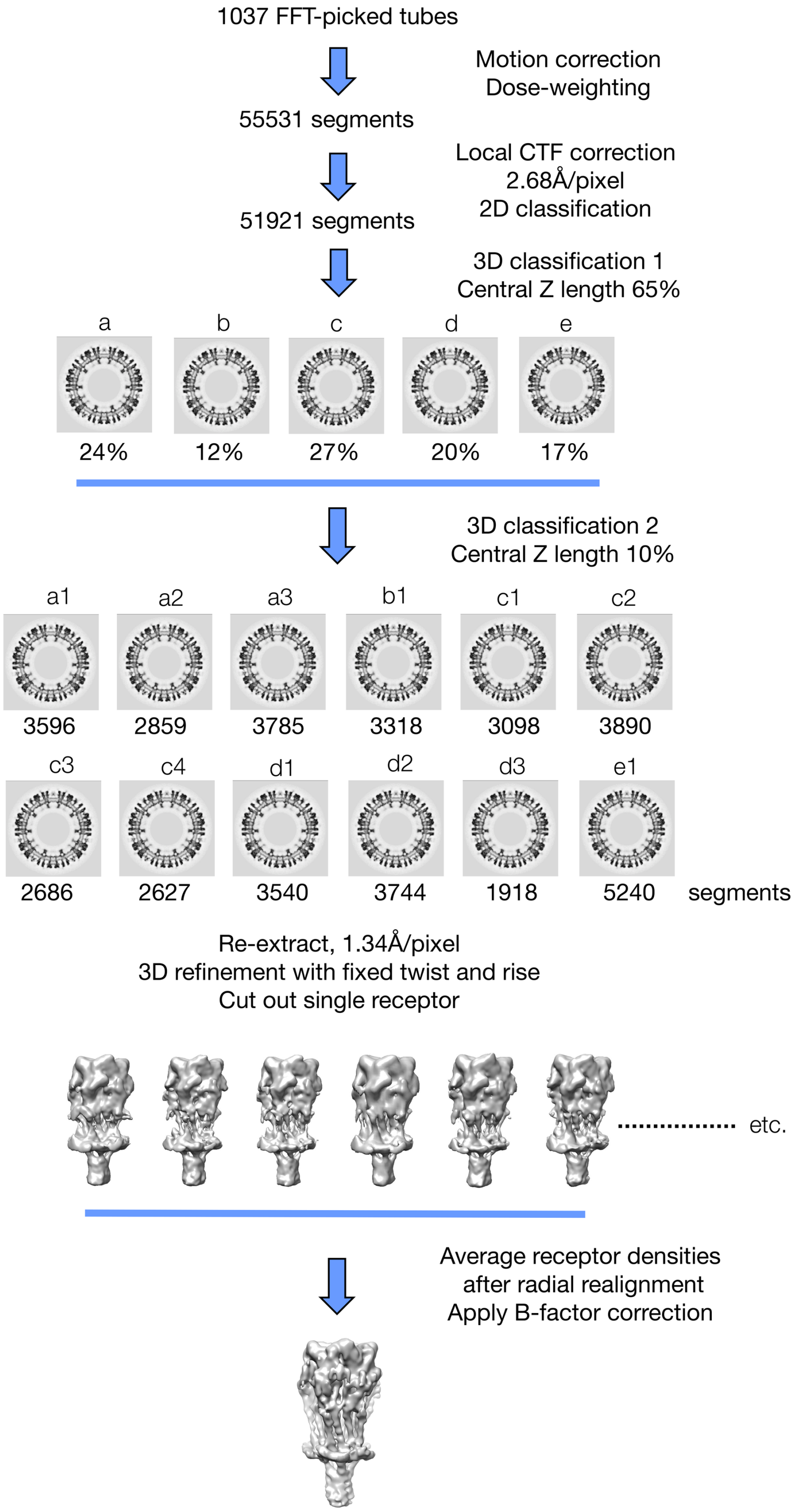

Figure S1 Image processing workflow to determine the structure of a single receptor from (-16,6) tubes. A regularization parameter T=10 was applied throughout to ensure significant contributions of the higher spatial frequencies. The same steps were applied to the (-17,5) tubes, based on 1374 micrographs. B-factor sharpening was not used except when applied the 'single-particle' maps (Fig. 1; Supplementary Figs. S4 and S5).

3D map	Twist (degrees)	Rise (Å)	Radius (Å)
a1	64.767	5.77	-
a2	64.765	5.78	-1.34
a3	64.767	5.78	-
b1	64.744	5.72	-4.02
c1	64.736	5.68	+2.68
c2	64.743	5.74	+1.34
c3	64.762	5.72	-
c4	64.748	5.72	-1.34
d1	64.764	5.77	-2.68
d2	64.741	5.77	-2.68
d3	64.754	5.75	-5.36
e1	64.753	5.75	+4.02

(*a*)

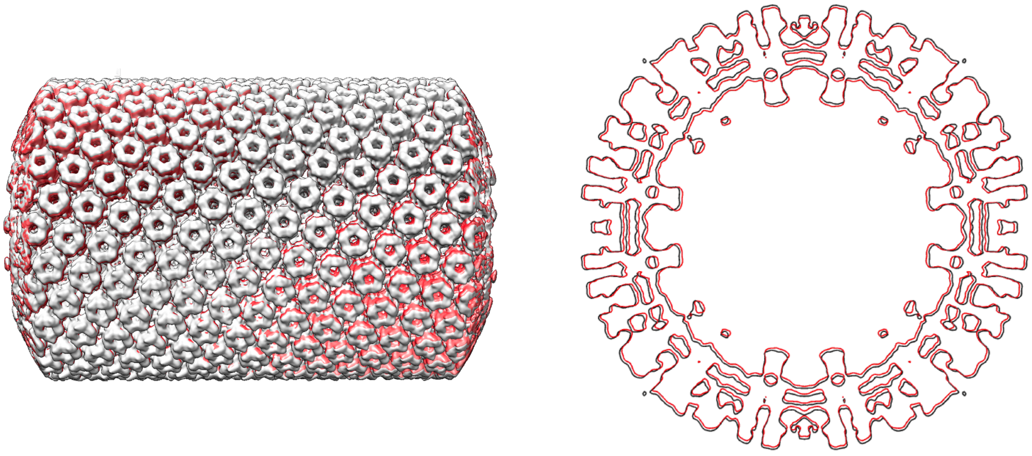

(*b*)

Figure S2 Properties of class averages. (*a*) List of parameters obtained for the set of 12 individual reconstructions ((-16,6) tubes; radial values are relative to map a1). Similar reconstructions were obtained by Fourier-Bessel analysis (Miyazawa *et al*., 1999), and classified according to an alternative notation. (*b*) Superimposed reconstructions from two class averages, viewed from above (left) and in cross-section (right). Differences in helical twist and rise, although small, are clearly visible when the reconstructions are superimposed; differences in tube radius are more significant (~4 Å in this example).

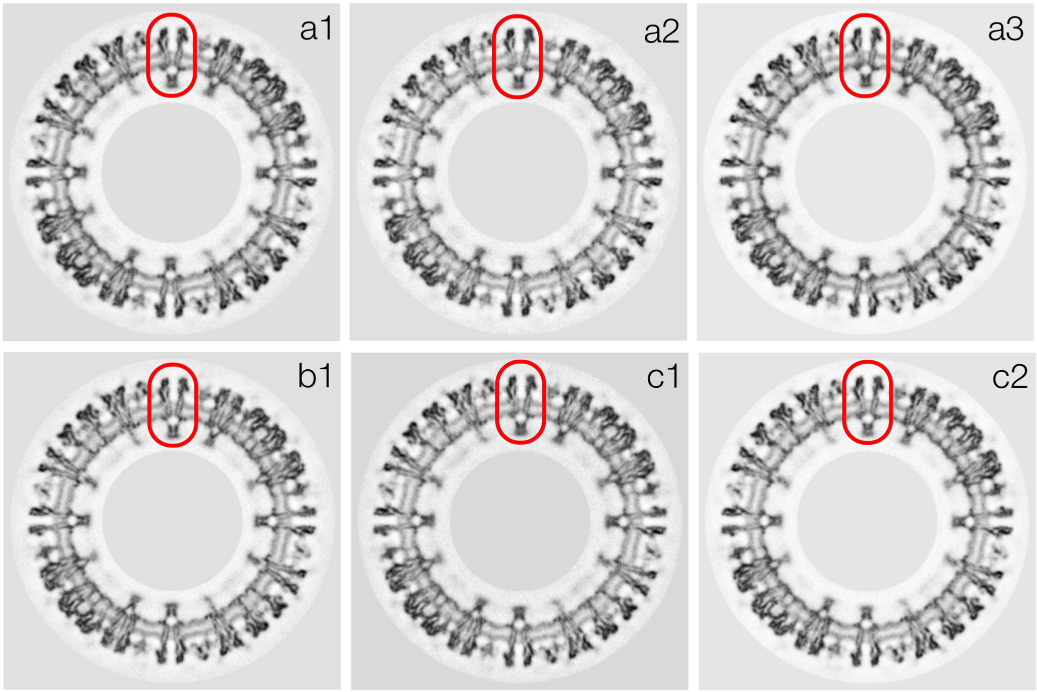

(*a*)

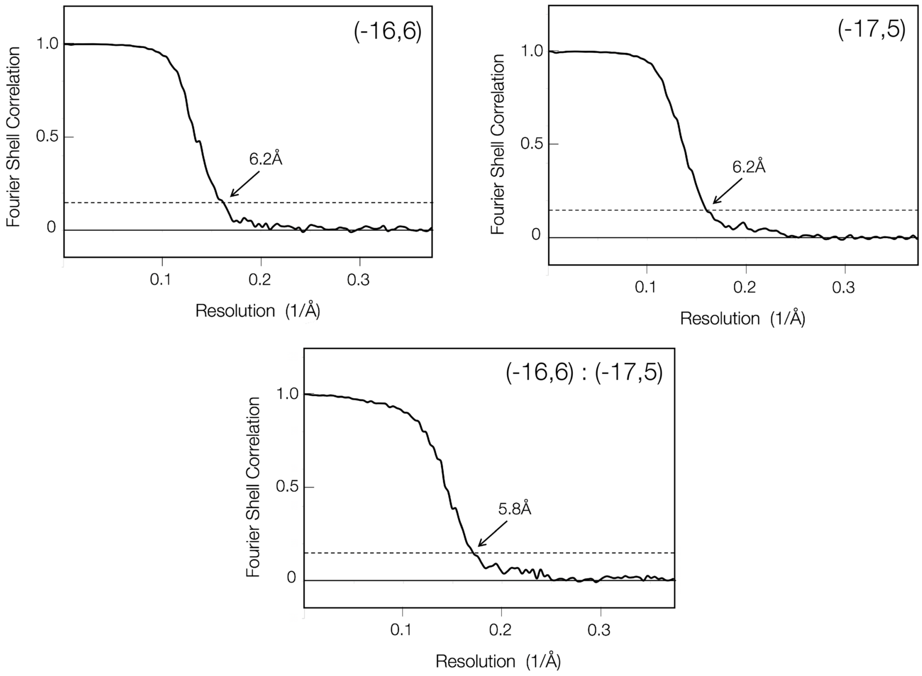

(*b*)

Figure S3 Averaging of densities comprising a single receptor. (*a*) Equivalent (radially aligned) regions from the set of 12 reconstructions (Supplementary Fig. 1) were cut out (red boxes) and averaged with weights proportional to the number of contributing segments. Shown are sections through reconstructions comprising a (-16,6) half-set. (*b*) Fourier shell correlation curves comparing half-set averages from the cut-out volumes in each helical family (upper), and the (-16,6) average with the (-17,5) average (lower). The resolutions are 6.2 Å for the family averages and 5.8 Å for the full average, estimated by the FSC = 0.143 threshold.

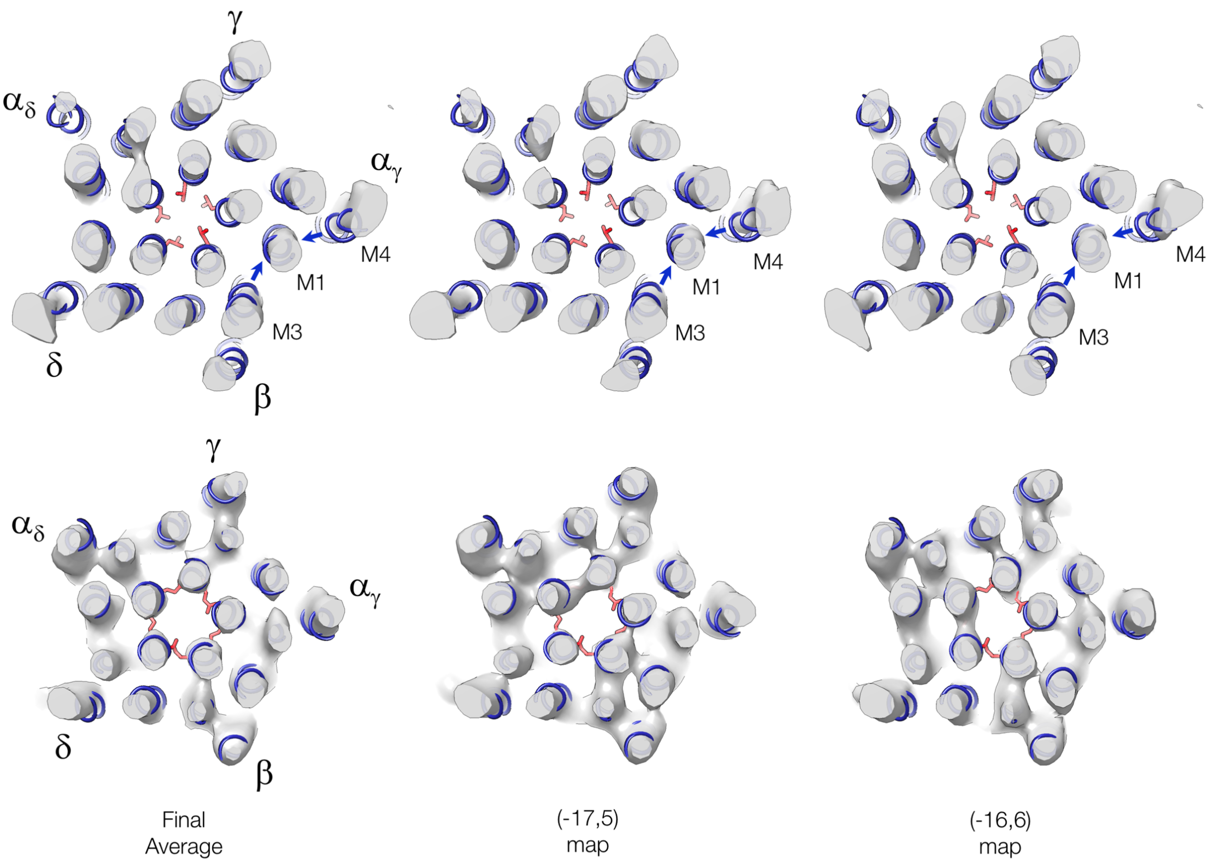

Figure S4 Comparison of the TM helix arrangement determined in this study with that of the solubilised protein (Rahman *et al*., 2020). Shown are slabs through the three density maps encompassing the 9' and -1' positions (residues in red), so identifying the outer (upper) and inner (lower) leaflets of the bilayer. In all cases, the model of the solubilized protein (PDB entry 6uwz) matches quite well the densities in the inner leaflet of the bilayer, but deviates substantially from the densities in the outer leaflet. The discrepancies in the outer leaflet reflect contraction in the model of the M4-M1 and M1-M3 interhelical spacings (arrows). Similar contractions occur in all five subunits, and they are equally apparent in the (-17,5) and (-16,6) maps as in the final average map. This effect cannot be due to magnification variance (Rahman *et al*., 2020), but is likely to have arisen because cholesterol needs to be present to stabilise and maintain the correct transmembrane architecture in the outer leaflet of the bilayer. Since cholesterol has been extracted from the protein that was used to derive the model, its stabilizing influence - and hence fidelity of the model - has been lost. A consequence of the contraction is that the upper portions of the M2 helices are drawn closer to the central axis, destroying the tapered shape of the pore which characterizes ACh receptors in their native membrane setting (Fig. 1*d*; Miyazawa *et al*., 2003; Unwin & Fujiyoshi, 2012).

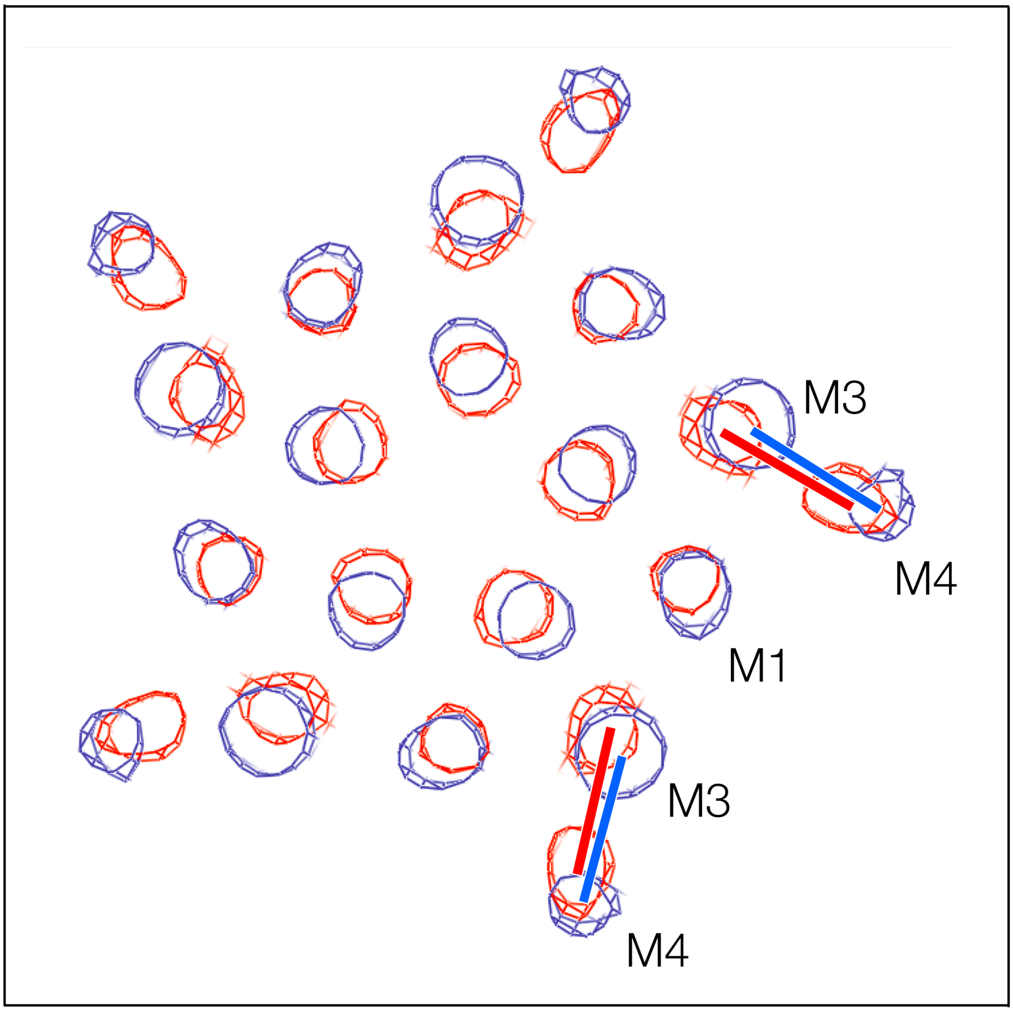

Figure S5 Sections at the level of the outer phospholipid headgroups comparing the TM helix densities from the membrane-bound protein (blue) with those from the solubilized protein (red), after five-fold averaging. The contraction at the lipid interface, associated with the solubilized protein, only implicates the cholesterol-occupied region M4-M1-M3; the separation M3-M4 (equal bars), where no cholesterol was detected, does not change. As the figure also indicates, there is an overall inward displacement of all the helices by ~2 Å at this level. The densities for the solubilized protein were derived from the deposited map EMD_20928. The bar length corresponds to 11.3 Å.

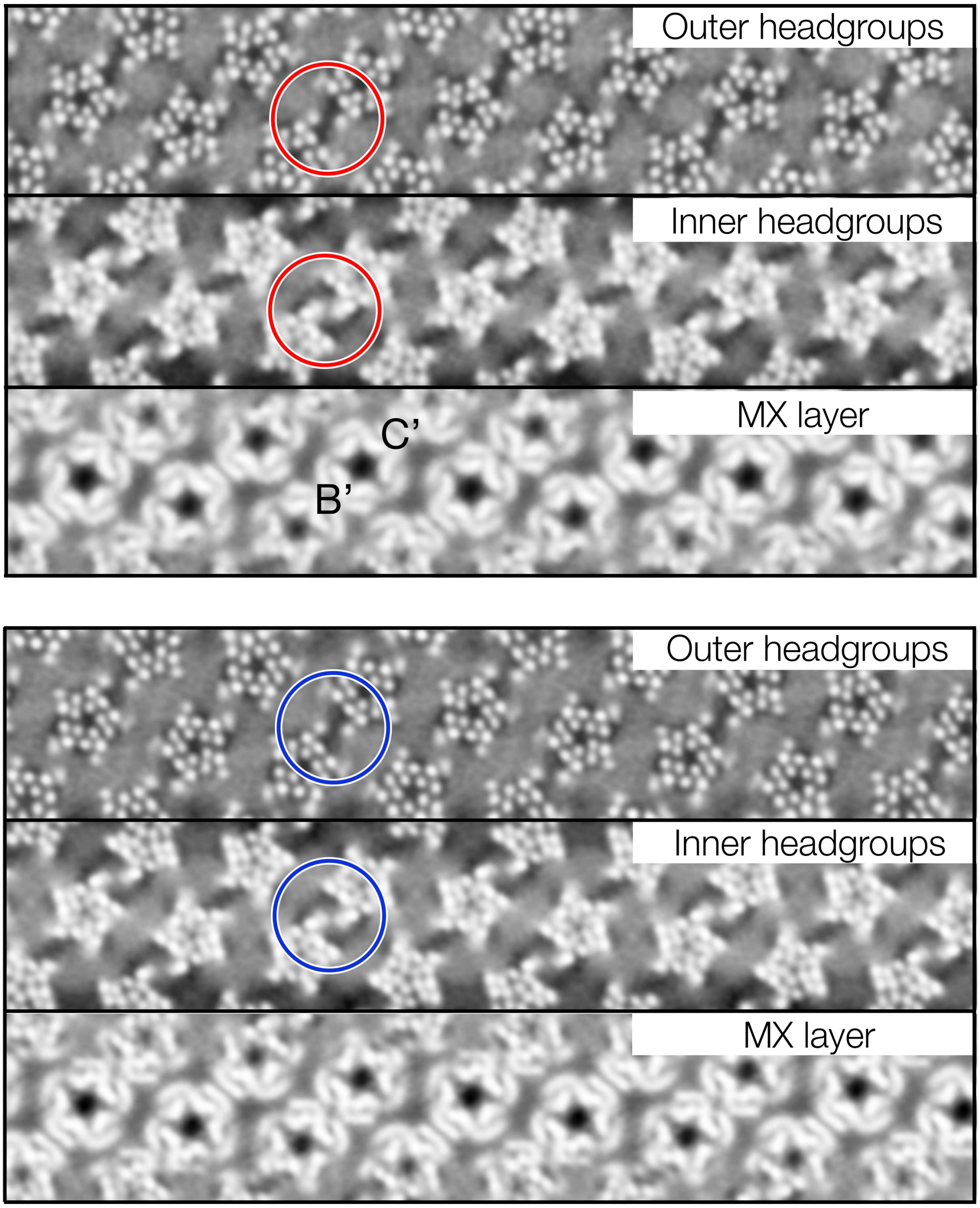

Figure S6 Comparison of equivalent sections through the outer and inner phospholipid headgroups and the underlying MX layer in the two helical families ((-17,5), top; (-16,6), bottom). The δ-δ microdomains are slightly enlarged in the (-16,6) tubes (blue circles), compared with those of the (-17,5) tubes (red circles), possibly reflecting a slightly higher cholesterol content. Labels B’ and C’ in the MX layer identify the MX interfaces underlying microdomains B and C. The MX (α_γ-α_γ) interface at C’ links the δ-δ dimers to each other. The C/C’ link is likely to be responsible for the longer range cooperative gating activity observed by Schindler *et al*. (1984) because it brings together neighbouring C loops shaping the α_γ ACh binding sites, which on ACh activation are drawn apart (Unwin & Fujiyoshi, 2012).
